# Bioinspired Granular Media Friction Pad: A Universal System for Friction Enhancement on Variety of Substrates

**DOI:** 10.3390/biomimetics7010009

**Published:** 2022-01-04

**Authors:** Halvor T. Tramsen, Lars Heepe, Stanislav N. Gorb

**Affiliations:** Department of Functional Morphology and Biomechanics, Zoological Institute, Kiel University, 24118 Kiel, Germany; htramsen@zoologie.uni-kiel.de (H.T.T.); lheepe@zoologie.uni-kiel.de (L.H.)

**Keywords:** tribology, soft matter, jamming transition, bioinspired surfaces, anti-slip systems, contact mechanics

## Abstract

The granular media friction pad (GMFP) inspired by the biological smooth attachment pads of cockroaches and grasshoppers employs passive jamming, to create high friction forces on a large variety of substrates. The granular medium inside the pad is encased by a flexible membrane which at contact formation greatly adapts to the substrate profile. Upon applying load, the granular medium undergoes the jamming transition and changes from fluid-like to solid-like properties. The jammed granular medium, in combination with the deformation of the encasing elastic membrane, results in high friction forces on a multitude of substrate topographies. Here we explore the effect of elasticity variation on the generation of friction by varying granular media filling quantity as well as membrane modulus and thickness. We systematically investigate contact area and robustness against substrate contamination, and we also determine friction coefficients for various loading forces and substrates. Depending on the substrate topography and loading forces, a low filling quantity and a thin, elastic membrane can be favorable, in order to generate the highest friction forces.

## 1. Introduction

For the maximization of friction forces on an unknown substrate, we recently introduced the granular media friction pad (GMFP) [1] that combines the advantages of an extremely soft, liquid-like material when coming into contact with the substrate with a rigidified material exhibiting high internal friction when pressed onto the substrate and sliding along the substrate. This is achieved employing a very thin elastic membrane that encases a loosely filled granular media which undergoes the jamming transition [2,3] when a load is applied by pressing the GMFP against the substrate.

**Figure 1:**
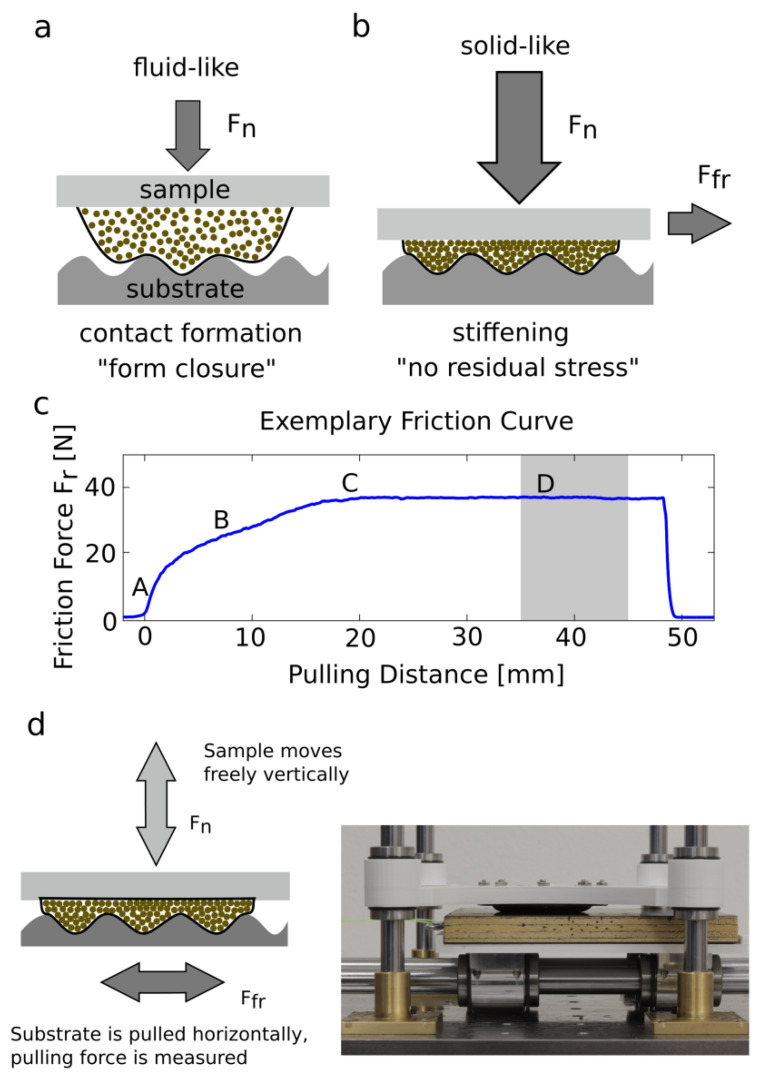
Granular media friction pad (GMFP) [1]. (**a**) Fluid-like state upon coming into contact. (**b**) Solid-like state under high normal load. (**c**) Exemplary friction curve. (**d**) Experimental setup.

When coming into contact with a surface, the granular media behaves like a fluid [4,5] and flows around the substrate asperities (see Figure 1a). After applying normal load, the granular media undergoes the jamming transition [2,3], thus creating high friction forces by mechanical interlocking as well as high internal friction of the granular media [6,7,8,9,10,11] (see Figure 1b). Upon removing the normal load from the GMFP, the granular material returns to its fluid-like state and the GMFP can be removed from the substrate without requiring high pull-off forces. The thin flexible membrane encasing the granular media contributes to maximizing contact area for adhesion-mediated friction [12,13,14], as well as to energy dissipation during sliding due to the stretching of the membrane. The combination of normal force pressing the GMFP onto the substrate and the encasing membrane is sufficient for the GMFP to undergo the jamming transition. No active control mechanism, such as applying a vacuum to induce and control the jamming of the granular material [4,15,16,17,18,19,20] is needed. When not in contact with a substrate or under a static load, the function of the membrane is mostly to encase the granular media. During sliding, however, the membrane plays a vital role. The extent, to which the granular material can dissipate energy by jamming, and the dilatation, due to shear and normal load, are partly determined by the elasticity of the membrane resulting from membrane thickness and modulus. To characterize the friction properties of a system, often the static and the dynamic friction coefficients are used. These two properties describe the relationship between the normal force acting on the system and the resulting friction force from a shearing motion of two contacting bodies. However, it is equally important, (1) how the two bodies come into contact and (2) how the GMFP adapts to the substrate topography since this greatly influences the resulting contact area between the two bodies, the basic requirement for the friction generation in soft materials. What is more relevant for the generation of friction on any substrate and any loading condition: the sample being very soft, to increase adaptability and contact formation, or the sample, being very stiff to increase energy dissipation by the granular medium, as well as by the deformation of the membrane?

The stiffness of the GMFP is determined by two factors, the granular media, and the encasing membrane. For granular media, detailed analyses on the jamming transition and interparticle friction depending on particle type and shape have been conducted [7,17,21,22,23,24], and knowledge about particle selection for maximizing friction exists. For membrane materials used in granular jamming systems, studies investigating the material’s influence on the behavior of the system have been conducted [25]. However, regardless of the individual components’ properties, frictional systems always have to be examined as a whole. The interplay of components and the resulting properties are as important as the components’ properties themselves. How does the filling quantity of the granular media influence the stiffness of the GMFP and the friction coefficient through an earlier onset of granular jamming when densely packed? How does the thickness or the elastic modulus of the membrane change the adaptability to substrate asperities or the amount of energy dissipation during stretching?

In this paper, we will investigate the effect that changing the bioinspired GMFP’s stiffness has on its friction properties by examining the resulting contact area and the dynamic friction coefficients on three different types of substrates. We systematically varied the filling quantity and the stiffness *D* of the membrane, being *D* *~* *Et*^3^ with the membrane’s elastic modulus *E* and its thickness *t.* The interplay between the elastic behavior of the membrane and the deformation of the granular medium was analyzed experimentally and numerically in the first publication, where details of the functional mechanism were studied [1]. In the present paper, we focus more on the applied aspects of the GMFP. The idea was to test, how this system behaves in a real-world situation and how good is the chance for using this system in real applications. That is why three extreme scenarios of substrates were chosen: smooth, rough, and covered with loosened particles. The main goal of this paper is a further exploration of functional mechanisms and adaptation of previous research to the case of real application, for example as a foot for walking robot that should function well on the wide range of challenging substrates.

## 2. Materials and Methods

We systematically investigated the stiffness of the bioinspired granular media friction pad by varying the filling quantity, the thickness of the encasing membrane, as well as the membrane modulus. Two different types of experiments were conducted (Figure 1). The first experiment illustrates the static contact area for different normal loads *F_n_* on a smooth glass substrate contaminated by one large particle. For the second experiment, the GMFP’s dynamic friction coefficient on three different substrates at different normal loads is measured.

The reference granular media friction pad (GMFP) [1], of which one parameter is varied at a time, consists of a 0.45 mm thick silicone membrane (Dragon SkinTM 30, Smooth-On, Inc., Macungie, PA, USA) which is placed flat on a 3D-printed sample holder and screwed down with an adjusting washer (40 mm × 50 mm × 1 mm). Then, 1.7 g of ground coffee (Gold 100% Arabica, Markus Kaffee GmbH & Co. KG, Weyhe, Germany) is pushed behind the membrane through an access hole in the sample holder. The reason for using coffee was because, in the preliminary experiments [1], coffee was empirically found to be the best filler among 20 other organic and mineral media. Before each individual experiment, the sample is put on the substrate and swayed back and forth several times to ensure homogenous particle distribution and negate any effects of previous measurements.

In each subset of experiments, one of the following parameters is varied at a time:

- filling quantity (1 g, 1.7 g, 2.7 g, 3.7 g),
- membrane thickness (0.15 mm, 0.45 mm, 1 mm, 2 mm),
- membrane modulus (Dragon SkinTM 10 with 0.15 MPa, Dragon SkinTM 20 with 0.33 MPa, and Dragon SkinTM 30 with 0.6 MPa and corresponding shore hardness of 10 A, 20 A, and 30 A).

All sample types were produced four times and each sample was tested for all normal loads on three different substrate types in random order.

To investigate the effect of the change in stiffness of the GMFP on the contact area as well as on the pad’s adaptability to conform around a large substrate asperity, a glass sphere (2.5 mm diameter) was placed onto a smooth glass substrate. The sample was lowered onto the substrate and loaded with the respective normal load *F_n_*. The contact area was visualized using total internal reflection. For each normal load, the sample was completely lifted off the substrate and the glass sphere repositioned. Normal load ranged from the sample weight itself (1 N) up to a maximum of 112.09 N.

To examine the friction coefficient of the granular friction pad, we obtained the dynamic friction coefficient for all samples at different normal loads. The experimental setup consists of vertical and horizontal shafts with freely running linear bearings holding substrate and sample (see Figure 1d). The substrate is pulled for 50 mm along the horizontal shafts at 1mm/s using a linear testing machine (Xforce HP 500 N, ZwickiLine, Zwick Roell, Ulm, Germany) that measures the pulling force. Directly above the substrate, the sample runs freely on the vertical shafts. To increase the normal load *F_n_*, additional weights can be placed on top of the sample.

The substrates (100 mm × 100 mm) were chosen to represent a wide variety of different substrate types: a smooth surface (the smooth side of wire mesh plywood), a rough/structured surface (the structured side of wire mesh plywood), and a smooth surface contaminated by particles (the smooth side of wire mesh plywood contaminated by 0.5 g of 1–2 mm large gravel particles). The substrates were cleaned with soap water and wiped with distilled water between experimental runs. For the runs with particle contamination, 0.5 g of the gravel particles was tossed onto the substrate to simulate random particle contamination.

An exemplary friction curve of a GMFP on a clean smooth substrate at a normal load *F_n_* = 19.36 N is shown in Figure 1c. The obtained friction curves of the GMFP can be divided into four parts. The steep incline in the friction force at the beginning of experiment (A) results from the initial shearing of the jammed granular particles inside the GMFP. When the GMFP is deformed more strongly, in addition to the shearing of the particles, the flexible membrane is being stretched, which leads to a lower incline of the friction force (B). The GMFP remains in static contact for a long time before maximum static friction force is reached (C) and dynamic sliding on all parts of the membrane sets in (D). The gray area represents the evaluated timeframe (10 s) for the averaged dynamic friction force *F_fr_* and resulting dynamic friction coefficient *µ_fr_*. At the end of the experiment, the sensor head returns to its starting position, at which point the elastic membrane allows the sample to relax mostly to its initial configuration.

## 3. Results and Discussion

To investigate the effect of stiffness on the bioinspired granular media friction pad for contact formation, as well as for friction coefficient, we varied filling quantity, membrane modulus, and membrane thickness individually.

To be able to both see the maximum contact area on a smooth substrate as well as demonstrate the GMFP’s ability to adapt to large substrate asperities, we visualized the contact area of the GMFP in static contact with a glass substrate. A 2.5 mm diameter glass sphere, which resembles the contamination of the gravel particles of 1–2 mm particle size for the dynamic friction experiments, was put between GMFP and substrate, and the pad was loaded with eight different normal loads. The contact area lights up by total internal reflection. In the resulting pictures, the glass sphere is marked by a red dot.

The dynamic friction coefficient was measured by pulling the sample over all three substrates (smooth, structured, and contaminated) at four different normal loads.

### 3.1. Filling Quantity

**Figure 2:**
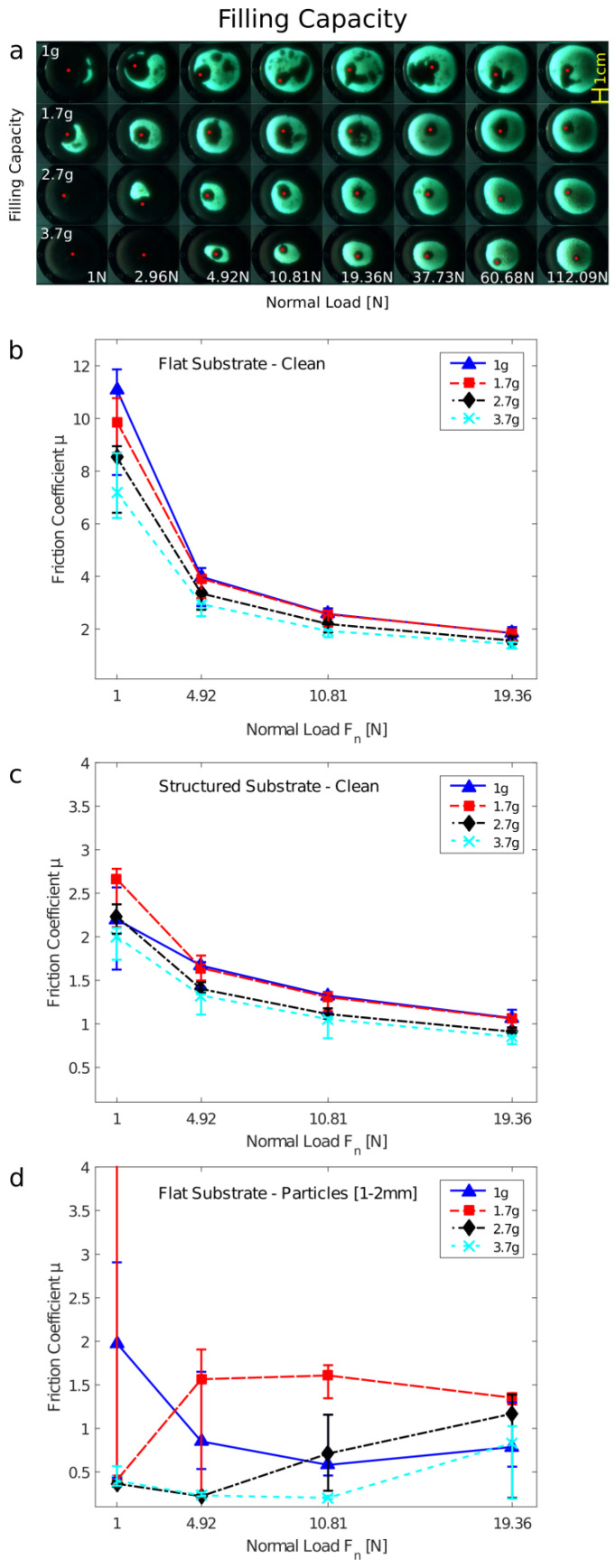
Variation of the filling quantity of the granular friction pad. (**a**) Visualization of the contact area on a glass sphere and smooth glass substrate. (**b**–**d**) Friction coefficient of the differently filled granular friction pad at different normal loads *F_n_*: (**b**) on a smooth substrate, (**c**) on a rough/structured substrate, (**d**) on a smooth substrate contaminated by 1–2 mm large particles.

By varying the filling quantity, we can change the elasticity of the GMFP. With less granular media inside, the GMFP becomes floppy and can easily adapt to the substrate. When the GMFP is filled more densely, an increase in pressure inside the granular media due to the stretching of the encasing membrane could result in higher energy dissipation from an earlier jamming transition at lower normal loads. While previously [1] the granular pad was filled with 1.7 g of ground coffee, we now examine four different capacities ranging from 1 g up to 3.7 g of granular material. The resulting static contact areas for the different filling capacities can be seen in Figure 2a. For higher filling capacities, higher normal loads are required for the GMFP to form around the glass sphere and to get into contact with the glass substrate. At high normal loads, all filling capacities achieve large contact areas with the substrate despite the glass sphere. However, the largest contact area with the substrate was still achieved on the less filled GMFPs with 1 g and 1.7 g granular media inside. The GMFP with the lowest filling quantity is only just about as high as the 2.5 mm glass sphere between sample and substrate. Thus, under all loading conditions, the glass sphere rests directly onto the sample holder, transferring most of the normal load. However, because of the low filling quantity, the nearly unstressed membrane can still achieve large contact areas with the glass substrate at low normal loads. For the 1.7 g filling quantity, the sample is large enough that normal loads are mostly transferred through the granular media, resulting in the largest contact areas overall.

The dynamic friction coefficients on the three different substrates can be seen in Figure 2b–d. For all filling capacities, the highest friction coefficient was measured on the smooth clean substrate (see Figure 2b). Due to the smooth substrate, contact area maximizes and adhesion-mediated friction strongly contributes to the occurring friction forces, resulting in very large friction coefficients at low normal loads [13,26]. Especially at the lower normal load, a trend towards higher friction forces at lower filling capacities is observed owing to a larger contact area. On the structured substrate (see Figure 2c), friction forces are lower compared to the smooth clean substrate. The lower filling capacities result in higher friction forces for all loading conditions. A clear difference in dynamic friction coefficients can be observed on the contaminated substrate (see Figure 2d). The GMFP filled with only 1 g of ground coffee is so flat that it sometimes collides with the contamination particles. Especially when the granular material is compacted even more at higher normal forces, the sample holder gets in direct contact with some of the larger gravel particles, resulting in lower friction force. The highly filled GMFP also achieves relatively low friction forces, since a high normal load is needed to press the pre-stretched membrane around the gravel particles on the substrate. The strange trend was visible for some filling capacities, especially for 1.0 g and 1.7 g, on the surfaces contaminated by particles. The reason for this behavior is the random distribution of differently sized particles on the substrate. This randomness leads to some specific behavior of the entire system in different individual experiments, depending on how many and which concrete size of particles are in contact.

### 3.2. Membrane Modulus

Previously [1], it has been shown that while the granular material contributes to the friction forces at the beginning of the pulling, the deformation of the membrane significantly contributes to the friction forces during the rest of the sliding motion. A stiffer membrane compresses the granular media more strongly than a softer membrane. Thus, the GMFP with the stiffer membrane will not adapt to surface asperities as easily, which results in a reduction of contact area [27]. However, the stiffer membrane will require more energy when deformed during sliding. Here we use silicone rubber with three different Young’s moduli of 0.15 MPa, 0.33 MPa, and 0.6 MPa.

**Figure 3:**
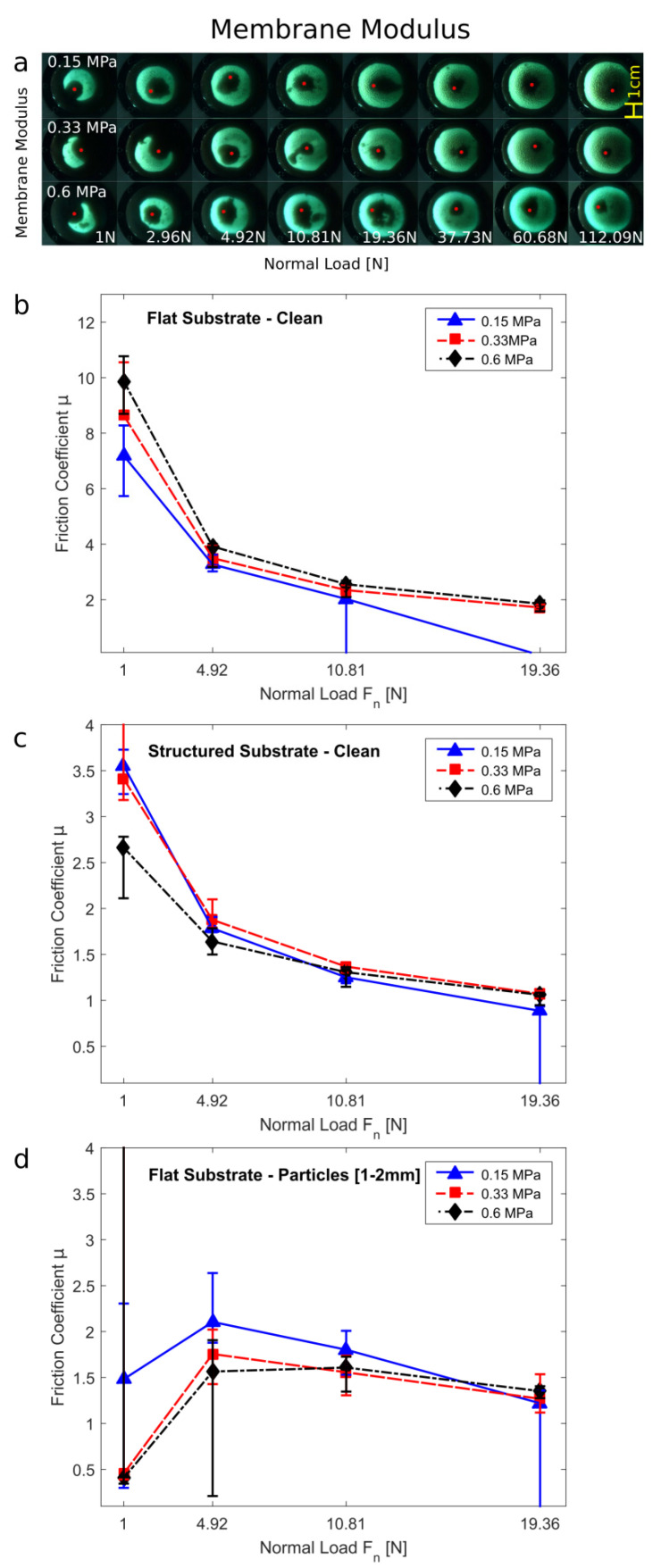
Variation of the membrane modulus of the granular friction pad. (**a**) Visualization of the contact area on a glass sphere and smooth glass substrate. (**b**–**d**) Friction coefficient of the samples at different normal loads *F_n_*: (**b**) on a smooth substrate, (**c**) on a rough/structured substrate, (**d**) on a smooth substrate contaminated by 1–2 mm large particles.

While the different elastic moduli strongly influence how the materials feel when manually handling it, the difference in static contact area for the different membrane moduli is very small (see Figure 3a). Only the softest 0.15 MPa sample shows a slightly larger contact area at lower normal loads due to its higher flexibility.

In the dynamic friction experiments, the highest friction forces were always observed on the smooth clean substrate (see Figure 3b). Interestingly, the stiffer the membrane, i.e., the higher the elastic modulus is, the higher the friction coefficient. Since the contact areas for all moduli are very similar, the difference in friction coefficient results from higher energy dissipation in stiffer membranes during their deformation when sliding over the substrate. At higher normal loads, the softest membrane ruptures and completely fails. Thus, a stiffer membrane is preferable, since contact formation and friction coefficient are similar, but the stiffer membranes are more resistant to failure. Under low normal loads, the stiffest membrane performed best on the smooth clean substrate. However, having even softer membrane results in higher adaptability to substrate asperities, which can not only be seen in Figure 3a for the low normal forces but also on the structured and the contaminated substrates (see Figure 3c,d). Here, the softer samples reach higher friction forces by coming into contact with the substrate despite the particle contamination.

### 3.3. Membrane Thickness

**Figure 4:**
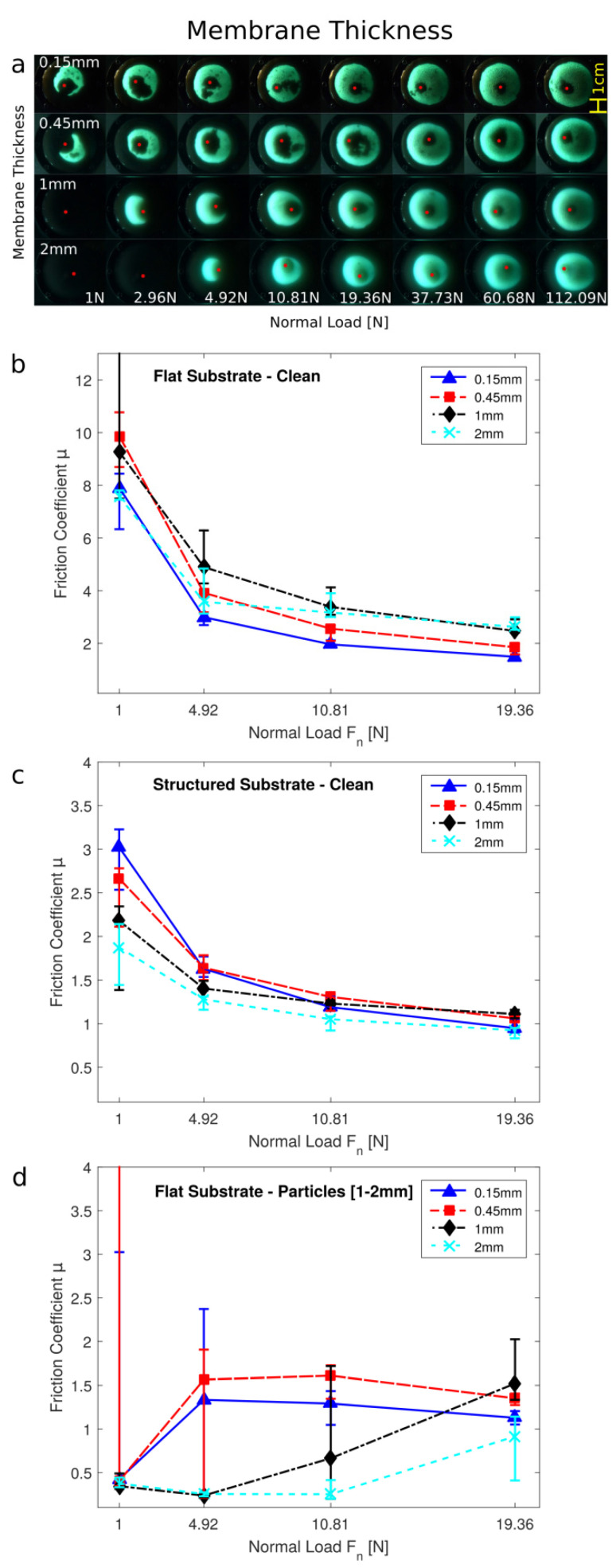
Variation of the membrane thickness of the granular friction pad. (**a**) Visualization of the contact area on a glass sphere and smooth glass substrate. (**b**–**d**) Friction coefficient of the samples at different normal loads *F_n_*: (**b**) on a smooth substrate, (**c**) on a rough/structured substrate, (**d**) on a smooth substrate contaminated by 1–2 mm large particles.

The stiffness of a GMFP’s membrane can not only be modified by changing its elastic modulus, but also by changing its thickness. Since the membrane thickness increases the membrane’s stiffness by the power of three instead of linearly like the modulus, a much more noticeable change in friction properties is expected. While the membrane thickness for the other tests was always 0.45 mm, here we also investigated thicknesses of 0.15 mm, 1 mm, and 2 mm. The change in membrane stiffness results in a big difference for the static contact area, as can be seen in Figure 4a. For the low loading conditions, the thinnest membrane achieves the largest contact area with the substrate. The thicker membranes need much higher loading forces to be pushed around the glass bead. Thus, for high robustness against contamination at low normal forces, a more flexible membrane is beneficial to facilitate the flowing of the granular particles.

A similar effect can be seen for the dynamic friction experiments (see Figure 4b–d). On the clean smooth substrate, the granular friction pad does not have to deform much to achieve large contact areas (see Figure 4b). Friction coefficients of the different membrane thicknesses are very close, with the thicker membranes achieving slightly higher friction coefficients at higher loading forces *F_n_*. On the clean structured substrate (see Figure 4c), a difference in friction coefficients can be seen at low normal loads. The thicker membranes do not adapt easily enough to create high friction forces at these low normal loads. Only at higher normal loads, similar friction forces as with a thinner membrane can be achieved. The largest differences between the four membrane thicknesses can be seen on the smooth substrate contaminated with particles (see Figure 4d). Due to the random distribution of the contaminating particles, even at low normal loads, the thinner membranes can sometimes conform around the particles and still achieve contact with the substrate during sliding, resulting in high friction forces. The thicker membranes are not flexible enough and only achieve contact at higher normal forces. The concept of the granular friction pad even works with the thickest 2 mm membrane, which is able to adapt around the particles and come into contact with the substrate but only at high normal forces.

### 3.4. Technological Implications

During the jamming transition of a granular medium, the dynamics become increasingly spatially heterogeneous and strongly reminiscent of the behavior of glass-forming liquids [28]. Such amorphous fluids become solid-like if either the temperature is lowered, or the density is increased (glass transition or jamming transition). The jamming transition was previously numerically modeled considering external forces and the orientations of contacts between particles, to compute all the interparticle forces [1,29]. In principle, this phenomenon is well understood; however, there is a number of other interesting effects related to this phenomenon, such as phase separation in the heterogeneous medium [30] or friction/adhesion effects at the interface between granular medium and non-smooth substrate separated by a flexible membrane [1,31]. Both these types of effects have interesting technological implications. Small-amplitude high-frequency vibrations affect the size separation of particles of a granular material, which may open new ways not only for the separation of fluids from solids and solids from solids but also for controlled pattern formations during the synthesis of composite materials [30].

For maximizing friction forces of robotic legs on an unknown/unpredictable substrate, the granular media friction pad was recently introduced [1]. It consists of a thin elastic membrane encasing loosely filled granular material. On coming into contact with a substrate, the fluid-like granular material flows around the substrate asperities and achieves large contact areas with the substrate. Upon applying load, the granular material undergoes the jamming transition, rigidifies, and becomes solid-like. High friction forces are generated by mechanical interlocking on rough substrates, internal friction of the granular media, and the enhanced contact area caused by the deformation of the membrane. The crucial difference of this system from any other previously studied tribological system is that it can adapt to a large variety of dry substrate topologies. To further increase its performance on moist or wet substrates, the granular media friction pad was recently enhanced by structuring the outside of the membrane with a 3D hexagonal pattern [31]. In the present study, we obtained the most detailed data on the bioinspired artificial GMFP depending on different parameters of the system, which might open the ways for the technological implication of the knowledge from the biology of smooth adhesive pads and from physics of granular media to artificial robotic systems with enhanced ability to contact formation and generation of grip even on rather challenging substrates.

### 3.5. Biological Implications

The elastic modulus of the pad is controlled here by the filling quantity of the granular medium, membrane stiffness, and membrane thickness. In biological systems, the use of these three mechanisms for tuning the mechanical properties of the pad was previously reported. The study on morphology, ultrastructure, effective elastic modulus, and attachment properties of two different smooth-type insect pads has been done for two orthopteran species: *Tettigonia viridissima* (Ensifera) and *Locusta migratoria* (Caelifera) [32]. Similar to the GMFP studied here, both insect species have in principle a similar structural organization of their attachment pads. However, the details of pad material structure were found to be different in these two species. *L. migratoria* pads bear a thick sub-superficial layer of tanned (stiffer) exocuticle, as well as a higher density of rods filling inside the pad, whereas *T. viridissima* pads possess a thin flexible superficial exocuticle containing rubber-like protein resilin. The obtained experimental results demonstrated a clear correlation between the density of the fibers, the thickness of the superficial layer, compliance of the pad, and its adhesive properties [32]. The material structures and properties in organisms are related to the preferred ecological niches of species. The data obtained in the present study on the bioinspired artificial GMFP is the most detailed implication of the knowledge from the biology of smooth adhesive pads to the artificial system with enhanced ability to contact formation and generation of grip even on rather challenging substrates.

## 4. Conclusions

To summarize, we investigated the stiffness dependence of the bioinspired granular media friction pad on the friction coefficient by varying filling quantity, membrane modulus, or membrane thickness. We could show that the amount of granular media inside the GMFP is extremely important for contact formation since an excessive filling quantity, hindering unjammed motion without load, greatly reduces adaptability to substrate asperities. However, the filling quantity has to be sufficient to be able to compensate for the highest substrate asperities of the substrate. At higher normal loads, the difference in filling quantity becomes less apparent and only facilitates friction on strongly contaminated substrates. Higher pressure inside the granular media due to high filling quantity and the resulting stretching of the membrane does not outweigh the loss in contact formation and resulting adhesion-mediated friction at all loading conditions.

Membrane elasticity is shown to be an equally important criterion for maximizing friction. While the effect of changing the membrane modulus is less pronounced on both contact area and friction coefficient, although stiffer and more durable material is to be favored, a change in membrane thickness greatly modifies the properties of the GMFP. By using a thicker membrane, more energy is needed for the deformation during sliding. However, at low normal forces, on the structure and especially on the contaminated substrate, a soft and thin membrane results in much higher friction forces.

Thus, an ideal GMFP’s filling quantity should be as low as possible, and its membrane should be the thinnest and most elastic while still retaining enough robustness for all conceivable loading conditions.

## Data Availability

Data available on request from the authors.
